# Multiple suppression pathways of canonical Wnt signalling control thymic epithelial senescence

**DOI:** 10.1016/j.mad.2011.04.007

**Published:** 2011-05

**Authors:** Zoltan Varecza, Krisztian Kvell, Gergely Talabér, Gyorgy Miskei, Veronika Csongei, Domokos Bartis, Graham Anderson, Eric J. Jenkinson, Judit E. Pongracz

**Affiliations:** a Department of Medical Biotechnology, Institute for Immunology and Biotechnology, Faculty of Medicine, University of Pécs, Pécs, Hungary; b Institute for Biomedical Research, Faculty of Medicine, University of Birmingham, Birmingham, UK

**Keywords:** Wnt signalling, PKCδ, Thymic epithelium, Thymic atrophy

## Abstract

Members of the Wnt family of secreted glyco-lipo-proteins affect intrathymic T-cell development and are abundantly secreted by thymic epithelial cells (TECs) that create the specific microenvironment for thymocytes to develop into mature T-cells. During ageing, Wnt expression declines allowing adipoid involution of the thymic epithelium leading to reduced naïve T-cell output. The protein kinase C (PKC) family of serine-threonine kinases is involved in numerous intracellular biochemical processes, including Wnt signal transduction. In the present study, PKCδ expression is shown to increase with age and to co-localise with Wnt receptors Frizzled (Fz)-4 and -6. It is also demonstrated that connective tissue growth factor (CTGF) is a Wnt-4 target gene and is potentially involved in a negative feed-back loop of Wnt signal regulation. Down-regulation of Wnt-4 expression and activation of multiple repressor pathways suppressing β-catenin dependent signalling in TECs contribute to the initiation of thymic senescence.

## Highlights

► Active Wnt-4 receptors, Fz-4 and Fz-6 are up-regulated during initialisation of thymic atrophy. ► Inhibitory receptor Fz-6, a negative regulator of β-catenin dependent Wnt signalling is increasingly associated with PKCδ during initialisation of thymic ageing. ► One of the Wnt-4 target genes is connective tissue growth factor (CTGF). ► CTGF, a suppressor of canonical Wnt signalling is up-regulated during ageing. ► Decline of the thymic epithelial network is initiated by active suppression of Wnt signalling.

## 1 Introduction

During ageing of the immune system the gradual loss of naïve T-cells is associated with the rate of thymic adipose involution that correlates with significant destruction of the epithelial network. As impaired T-cell production leads to weakened immune responses, understanding the mechanism of thymic involution has high physiological and medical importance.

In our recent studies of thymic involution Wnt-4 secretion was significantly reduced in TECs while LAP2α expression concomitantly increased triggering epithelial–mesenchymal transition (EMT) and then pre-adipocyte-differentiation (Kvell et al., 2010).

As Wnt-4 is the primary regulator of FoxN1 expression and consequently TEC identity, understanding Wnt-4 signalling carries particularly high importance (Balciunaite et al., 2002). The difficulty of signalling studies, however, stems from the general complexity of Wnt pathways (Kuhl and Pandur, 2009). Wnt-4, for example, has been described as activator of both β-catenin dependent canonical (Lyons et al., 2004) and JNK/PKC dependent non-canonical (Cai et al., 2002; Du et al., 1995) signalling pathways that interact at multiple levels. Apart from specific, there are also shared signalling elements in Wnt pathways including the main cell surface receptors Frizzleds (Fz) (Schulte and Bryja, 2007) as well as intracellular signalling molecules including G proteins (Malbon et al., 2001), Dishevelleds (Dvl) (Kuhl et al., 2001; Schulte and Bryja, 2007) and PKCs α (Kuhl et al., 2001), ζ (Ossipova et al., 2003), and δ (Kinoshita et al., 2003). PKCδ appears particularly important as this serine-threonin kinase can phosphorylate and therefore activate Dvls (Kinoshita et al., 2003) to relay ligand induced signals towards down-stream elements of Wnt cascades.

From the ten known mammalian Fz receptors, Fz-4 (Lyons et al., 2004) and Fz-6 (Lyons et al., 2004) have been confirmed to bind Wnt-4. Interestingly, while Fz-4 is an activator of the β-catenin dependent canonical pathway, signals from Fz-6 inhibit β-catenin dependent target gene transcription (Golan et al., 2004) indicating that regulation of Wnt-4 signalling might also begin at receptor level in the thymus.

As thymic involution is a complex physiological process and appears to be initiated by suppression of Wnt signals, understanding of receptor associated regulatory mechanisms can lead to target molecule recognition in the quest for re-juvenate the ageing thymus. To investigate the hypothesis, TECs of young and ageing adult Balb/c mice as well as a thymic epithelial cell line, TEP1 were used in the studies. Our experiments demonstrate that expression of Wnt receptors increase with age and that Frizzleds co-localize with signalling molecules PKCδ and Dvl. Examination of Wnt-4 target gene expression provides evidence for the existence of additional negative regulatory loops suppressing β-catenin dependent signalling that aids repression of thymic epithelial maintenance and provides opportunities for EMT and consequent adipocyte type differentiation.

## 2 Materials and methods

### 2.1 Cell culture

Tep1 (thymic epithelial) (Tanaka et al., 1993), 293 and Phoenix (PHX) human kidney epithelial cell lines were cultured in DMEM (Sigma–Aldrich) supplemented with 10% FCS and 100 μg of penicillin and streptomycin (Lonza Walkersville, Inc.).

### 2.2 Primary thymic epithelial cells

Balb/c mouse thymi (1 and 9 months) were the source of primary cell material. CD45^−^ EpCAM1^+^ TECs were isolated using magnetic cell sorting (Miltenyi Biotech) (purity was regularly above 90% – data not shown). Purified EpCAM1^+^ TECs were cultured in medium alone, or in medium supplemented with Wnt4.

### 2.3 Antibodies

For western blot analysis rabbit polyclonal anti-PKCδ (C-17), anti-Dvl(C-19) (Santa Cruz), anti-Fz-6 and anti-Fz-4 (R&D systems Inc.) antibodies were used as primary and HRP-conjugated anti-rabbit and anti-goat (Santa Cruz) were used as secondary antibodies. For fluorescent microscopy studies primary Abs were anti-PKCδ (658-676) pAb (Calbiochem) (1:100), anti-PKCδ (C-17) (Santa Cruz) (1:100), anti-Fz-4, anti-Fz-6 (R&D systems Inc.) (1:100) and FITC labelled anti-EpCAM1 (clone G8.8) (American Type Cell Culture Collection)(1:50) and anti-Ly51-PE (BD Pharmingen) (1:50) antibodies. Secondary antibodies were NorthernLights donkey anti-goat IgG-NL493 and NorthernLights donkey anti-rat IgG-NL557 and anti-rat and anti-rabbit IgG-NL663 (all from R&D Systems Inc.). (Dilution factor for all secondary antibodies was 1:200).

### 2.4 Histology using fluorescent antibodies

Frozen thymic sections (9 μm thick) were fixed in cold acetone for 10 min, then dried for 15 min and rehydrated and blocked using 5% bovine serum albumin (BSA in PBS for 20 min) before staining with the appropriate antibodies. The primary antibody was applied at appropriate dilution in 100 μl on all sections for 30 min followed by 3 washing steps with PBS for 5 min each. Secondary Ab was applied for 30 min followed by 3 × 5 min wash with PBS as above. PBS-glycerol 1:1 mix was applied before covering with slide covers. The sections were analysed by an Olympus BX-61 Fluorescent microscope or by Olympus Fluoview 300 confocal microscope using the Olympus Fluoview FV1000S-IX81 software.

Staining controls were the following: primary Ab with no secondary Ab, no primary just secondary Ab and irrelevant primary Ab for isotype control in combination with secondary Ab. All the stainings were repeated for a minimum of three times.

### 2.5 Subcloning of Wnt-4 and full length PKCδ

Wnt-4 was purchased and subcloned from a commercially available vector (Origene), while the full length PKCδ was a kind gift of Jae-Won Soh, Tnha University, Korea. Both Wnt-4 and PKCδ sequences were subcloned into the MIGRI retroviral vector (gift from W.S. Pear, Department of Pathology and Laboratory Medicine, University of Pennsylvania, PA). Retrovirus was produced by transfecting the plasmid DNA into the Phoenix packaging cell line (American Type Cell Culture Collection) using Lipofectamine 2000 (Invitrogen).

### 2.6 Transient transfection of siRNA PKCδ

siRNA specific for PKCδ was supplied by Santa Cruz. Tep1 cells were grown to 80% confluency and then siRNA and control siRNA was delivered using Lipofectamine according to manufacturer's instruction.

### 2.7 Cell sorting

Tep1 cells were infected with recombinant retroviruses encoding GFP, Wnt-4-GFP or wild type- PKCδ-GFP then sorted based on GFP expression by FACSVantage Cell Sorter (BD). GFP positive cells were cultured further under conditions described in Section 2.1.

### 2.8 Reverse transcription polymerase chain reaction (RT-PCR) and quantitative RT-PCR (Q-RT-PCR)

RT-PCR was conducted as described previously (Kvell et al., 2010). Q-RT-PCR was performed using SYBR Green Q-RT-PCR reagents and random hexamer primers (Applied Biosystems) as recommended by the manufacturer using an ABI Prism 7900HT sequence detection system. Threshold cycles (*C*_T_) for three replicate reactions were determined using Sequence Detection System software (version 2.2.2), and relative transcript abundance was calculated following normalization with a β-actin PCR amplicon. Quantitation of Q-RT-PCR products were based on a standard curve generated from untreated TEP1 cell line gene expression. PCR primer sequences are listed in Table 1.

### 2.9 PKCδ activation assay

Tep1 cells were lysed in RIPA buffer supplemented with protease and phosphatase inhibitors (Sigma–Aldrich) and immunoprecipitated with rabbit anti-PKCδ (658-676) pAb (Calbiochem) and protein G resin (Sigma–Aldrich) overnight at 4 °C. Kinase assay was performed using an HTScan PKCδ Kinase–assay Kit (Cell Signaling Technology Inc.) with biotinylated substrate peptide in the presence of diluted PKCδ. Active PKCδ kinase GST fusion protein was supplied to the kit as positive control. PKCδ specific activity was quantified in a colorimetric ELISA Assay using 96-well streptavidin-coated plates (Institute of Isotopes, Budapest, and Soft Flow Hungary Ltd., Hungary). Phosphorylation level of biotynilated substrates from each kinase reaction mix were measured using a rabbit anti phosphoSer/Thr-antibody (1:1000) (provided with the kit) detected by a HRP-labelled anti-rabbit (1:1000) (Santa Cruz) in the presence of TMB substrate. Optical density (absorbance) was read in an iEMS Reader MF V2.9 (Thermo Scientific, Waltham, MA) spectrophotometer using a bi-chromatic measurement system at 450 nm and 620 nm as reference.

### 2.10 Purification of proteins from cell membrane and cytosol

Tep1 cells (1 × 10^6^/condition) were treated with Wnt-4 and control supernatants for 30 min then cells were pelletted and cytosolic and membrane proteins were isolated as described previously (McMillan et al., 2003). Proteins of cytosolic and membrane fractions were separated in 10%SDS PAGE, blotted, blocked in 3% fat-free milk and probed for PKCδ protein. To ensure equal loading protein levels were visualised by Ponceau Red staining, when proteins were entering the separating gel.

### 2.11 Immunoprecipitation and Western blotting

Cell lysates were immunoprecipitated using anti-Fz-4 and anti-Fz-6 antibodies (R&D systems Inc.) and protein G resin (Sigma–Aldrich) in the presence of protease and phosphatase inhibitors (Sigma–Aldrich). Proteins were resolved in 10% SDS–PAGE, blotted onto nitrocellulose membranes, then blocked in buffer containing 3% fat-free dried milk and probed for the proteins of interest with primary then in the appropriate HRP-conjugated secondary antibodies. Proteins were visualized by enhanced chemiluminescence (Pierce) according to the manufacturer's instructions in a FUJI LAS4000 image station.

### 2.12 Microarray

Tep 1 cells were washed for one hour in FCS free medium then the cells were treated for 1 hr with Tep1 supernatants of control and Wnt4 over-expressing Tep1 cell lines, respectively. Following incubation cells were collected, RNA was purified, then mRNA were amplified and microarrays were performed in the Centre for Genomics, University of Debrecen, Hungary. Gene expression changes beyond 0.5*x*/2-fold cut off criteria were accepted. Genes demonstrating the largest fold differences were validated by qPCR then tested in an additional three independent experiments. A list of genes upregulated in the microarray experiment and selected for further analysis can be viewed in Supplementary Table 1s. In the present study CTGF (Connective Tissue Growth Factor) was selected as a read-out gene, as it reproducibly changed expression levels in additional experiments.

### 2.13 Statistics

Experiments were repeated at least three times and statistical significance was determined using the Student's *t*-test. *P* < 0.05 denoted statistically significant.

## 3 Results

### 3.1 Fz-4 and Fz-6 levels are affected by age

**Fig. 1:**
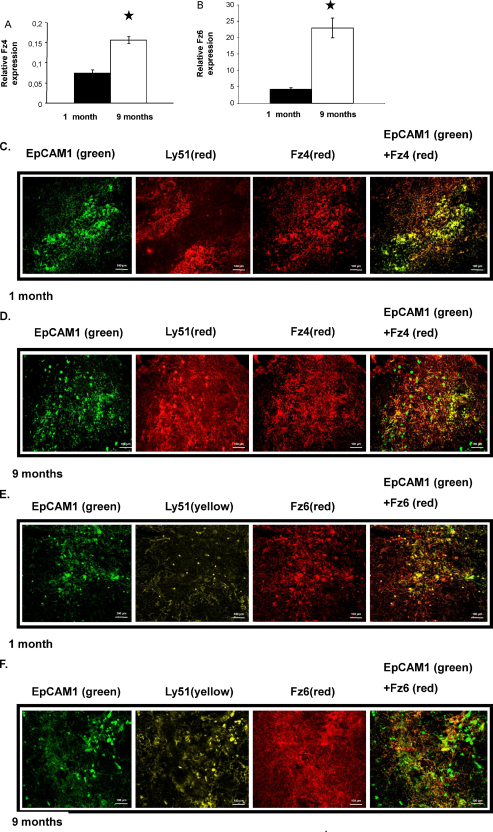
Fz-4 and Fz-6 expression during thymic senescence. (A and B) Q-RT-PCR analysis of Fz-4 and Fz-6 expression in young (1month) and ageing (9 months) mouse thymic epithelium. Data was normalised to β-actin. Statistically significant differences are marked by asterisks. (C–F) The expression level and staining pattern of Fz-4 and Fz-6 was also assayed by histology using anti-Fz-4-NL663 and anti-Fz-6-NL663 antibodies, respectively. Thymic morphology was displayed via staining with anti-EpCAM1-FITC and anti-Ly51-PE TEC markers. Size marker is shown in the corner of the figure. Characteristic stainings are shown from a minimum of five repeats.

Expression levels of Wnt-4 receptors, Fz-4 and Fz-6 were analysed in thymi of 1 month and 9 month old mice. The two age groups were selected specifically, as 1 month old mice are considered to be young adults where thymic involution has not yet begun. At the age of 9 months age associated morphological changes are not substantial yet, but characteristic age associated molecular and morphological changes can already be detected. This is not surprising as the detection of small lipid droplet-expressing cells in the perivascular space of *FoxN1Cre mice* has recently been reported as early as 3 months of age (Youm et al., 2009). Q-RT-PCR analysis of purified EpCAM1^+^ TECs of 1 and 9 month old mouse thymi showed increased expression of Fz-4 and Fz-6 mRNA (Fig. 1a and b) by 9 months of age. Immunohistochemistry using Fz-4 and Fz-6 specific antibodies confirmed elevated levels of both receptor proteins (Fig. 1c–f) in ageing thymi. Additionally, differential expression pattern of Fz4 and Fz6 was also observed in the thymic medulla and cortex. While in the young thymus the medulla (EpCAM1^++^/Ly51^−^) was preferentially stained for Fz4 and Fz6, the cortex (EpCAM1^+^/Ly51^+^) only faintly stained for this receptor. In the 9 month old thymus the medulla is less pronounced and in contrast to the young tissue and the whole section including the cortex appears increasingly positive for both receptors.

### 3.2 Active receptor signalling is indicated by PKCδ translocation

**Fig. 2:**
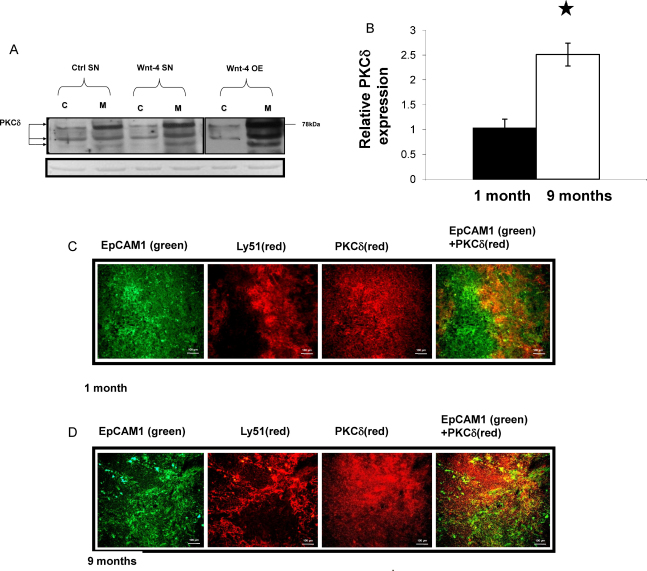
(A) Intracellular translocation of PKCδ following Wnt-4 treatment. Cytosolic and membrane proteins were separated from control, Wnt-4 treated and Wnt-4 overexpressing Tep1 cells. Western blot analysis demonstrated PKCδ translocation. Loading controls are shown below the Western blot as Ponceau red stained total protein. Representative blots and stainings are shown from three repeats. (B) Changes of PKCδ expression with age by Q-RT-PCR. TECs were purified from young (1 month) and ageing (9 months) mice. Data were normalized to β-actin housekeeping gene. Statistically significant differences are marked by asterisks. (C and D) Age associated changes in PKCδ expression by histology. Cryostate sections of 1 month and 9 months old mouse thymi were stained with anti- PKCδ− NL663, anti-EpCAM1-FITC and anti-Ly51-PE. The overlay of the staining pattern is also shown. Characteristic stainings are shown from a minimum of five repeats.

Active receptor signalling is invariably associated with modified phosphorylation of receptor associated signalling molecules. As protein phosphorylation depends on the activity of kinases, being an acknowledged activator of Dvl, PKCδ activity was investigated in Wnt-4 signalling. To test the involvement of PKCδ in Wnt-4 signal transduction, increased Wnt-4 presence was achieved by treatment using the supernatant of Wnt-4-transgenic thymic epithelial (Tep1) cells (Supplementary Fig. 1). Wild type Tep1 cells were exposed to SNs of control (Tep1-GFP) and Wnt-4 (Tep1-Wnt-4-GFP) cells for 1 h, then cytosolic and membrane fractions were isolated from cell lysates. Similarly to previous studies with Wnt-5a (Giorgione et al., 2003), Western blot analysis revealed that within one hour of Wnt-4 exposure PKCδ translocated into the membrane fraction (Fig. 2a) where the cleavage products (Kanthasamy et al., 2006) characteristic of active PKCδ were detected. Densitometric analysis of total and cleaved PKCδ demonstrated PKCδ activation (2 fold increase) upon Wnt-4 exposure. Additionally, increased membrane localisation of PKCδ was also detected (39 fold increase) in the Wnt-4-overexpressing cell line (Fig. 2a). As for Wnt-4 specific receptor expression, both Fz-4 and Fz-6 levels increased with age, therefore it was assumed that active receptor signalling might require more PKCδ during ageing. Indeed, apart from localisation of PKCδ to the membrane fraction (Fig. 2a), up-regulation of PKCδ was also detected at both mRNA (Fig. 2b) and protein level (Fig. 2c and d) in the ageing thymi. Interestingly, a characteristic cortico-medullary PKCδ pattern has also emerged. In both young and ageing thymi PKCδ was preferentially expressed in the cortex (EpCAM1^+^/Ly51^+^) (Fig. 2c and d).

### 3.3 PKCδ in Wnt-4 signalling

**Fig. 3:**
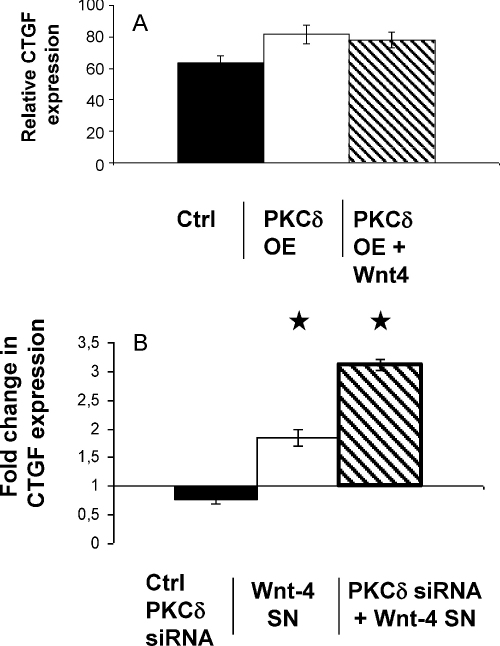
(A) Analysis of Wnt-4 target gene expression. Q-RT-PCR analysis of CTGF expression in control and Wnt-4 treated PKCδ over-expressing Tep1 cell line. (B) Q-RT-PCR analysis of CTGF expression in control and Wnt-4 treated in Tep1 cell lines pre-treated with siRNA for PKCδ. Data were normalized to β-actin. Statistically significant differences are marked by asterisks.

To specify the role of PKCδ in Wnt-4 signalling, it was necessary to identify a read-out gene that would reliably respond to Wnt-4 stimulus. Microarray (Supplementary Table 1) and subsequent Q-RT-PCR analysis of Wnt-4 exposed Tep1 cells identified connective tissue growth factor (CTGF) as such a target gene for Wnt-4 (Fig. 1s d). To investigate PKCδ involvement in Wnt-4 signalling, PKCδ activity was modified by overexpression of wild type PKCδ (Supplementary Fig. 2a–c) or by PKCδ gene specific silencing using commercially available siRNA for PKCδ (Santa Cruz) (supplementary Fig. 2d). Tep1 cells with increased or decreased PKCδ levels were exposed to control and Wnt-4 SNs for 1 hr, then CTGF expression was analysed. Surprisingly, although over-expression of PKCδ had no radical effect on Wnt-4 target gene transcription (Fig. 3a), even moderate down-regulation of PKCδ was able to significantly increase CTGF expression (Fig. 3b).

### 3.4 Co-immunoprecipitation of PKCδ, with Dvl, Fz-4 and Fz-6

**Fig. 4:**
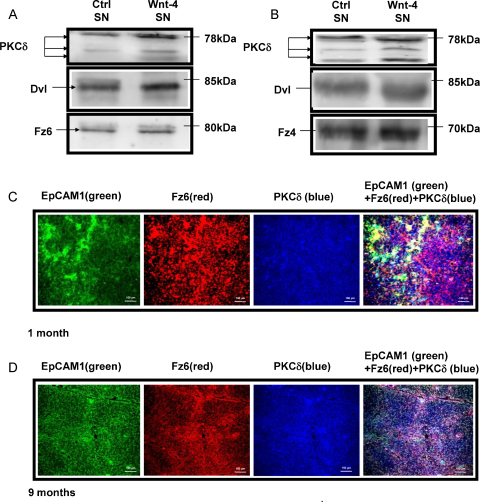
Analysis of Fz-4, Fz-6, Dvl and PKCδ co-localisation. (A and B) Western blot analysis following immunoprecipitation using anti-Fz-6-antibody (A) or anti-Fz-4-antibody (B) shows increased association of Fz-6 with PKCδ and Dvl upon Wnt-4 treatment. Representative blots are shown from two repeats. (C and D) Histology of 1 month and 9 months old mouse thymi using anti- PKCδ– NL663, anti-Fz-6-NL557 and anti-EpCAM1-FITC antibodies. The overlay of the three stainings is shown. Size markers are shown in the corner of all histology figures. Characteristic stainings are shown from a minimum of five repeats.

To investigate whether PKCδ can associate with Fz-s, Fz-4 and Fz-6 co-immunoprecipitation patterns were analysed. Tep1 cells were treated with control and Wnt-4 SNs then proteins were immunoprecipitated using anti-Fz-4 and –Fz-6 antibodies, and probed for PKCδ and Dvl. Immunoprecipitation revealed increased association of PKCδ and its active cleavage products with both Fz-4 (1.2 fold, 2 fold and 1.4 fold, respectively) and Fz-6 (1.4 fold, 1.5 fold and strongly detectable over non-detectable, respectively) upon Wnt-4 treatment (Fig. 4a). To find out whether PKCδ co-localises with Fz-6 in primary thymic tissue, immunohistochemistry was performed. Experiments demonstrated age dependent increase of both Fz-6 and PKCδ as well as co-localisation of Fz-6 and PKCδ staining (Fig. 4c and d). While in the young thymus Fz-6 and PKCδ co-localisation is more pronounced in the thymic cortex (Fig. 4c), in the ageing thymus it is the medulla that exhibits stronger staining for both proteins (Fig. 4d).

### 3.5 Increased expression of CTGF and Fz-8

**Fig. 5:**
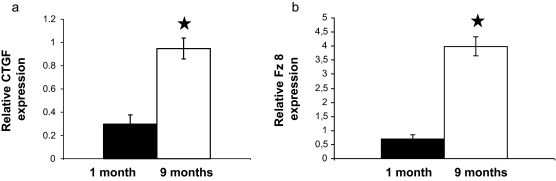
CTGF and Fz-8 expression during thymic senescence. (A and B) Q-RT-PCR analysis of CTGF and Fz-8 expression in young (1 month) and ageing (9 months) mouse thymic epithelium. Data was normalised to β-actin. Statistically significant differences are marked by asterisks.

While increased expression and activity of the Fz-6 receptor, a suppressor of the canonical Wnt signalling pathway explains some aspects of uneven target gene transcription following manipulation of PKCδ activity, parallel changes like up-regulation of Fz-4 also occur during ageing that might add to the complexity of the signalling process. Increase in Fz-4 levels in ageing mice correlated with increased CTGF gene expression (Fig. 5a). If Fz-6 that also increases during senescence is truly a suppressor of β-catenin signalling then CTGF expression should have decreased or remained unchanged as Fz-4 transmitted signals would have been quenched by Fz-6 signalling. To test the above hypothesis, we have considered the following: CTGF has recently been reported to negatively regulate canonical Wnt signalling by blocking β-catenin stabilisation via GSK3β activation leading to phosphorylation and consequent degradation of β-catenin (Luo et al., 2004), indicating that CTGF might be part of a negative feed-back loop. As CTGF is a secreted protein, expression of Fz-8 (Mercurio et al., 2004) a recently reported receptor for CTGF was analysed in purified TECs of 1 and 9 months old thymi using Q-RT-PCR reactions. As parallel with CTGF, Fz-8 mRNA levels increased (Fig. 5b) in ageing mice, while FoxN1 (data not shown) similarly to 12 months old mice reported previously (Kvell et al., 2010), a direct target of β-catenin dependent Wnt-4 signals (Balciunaite et al., 2002) was undetectable indicating the existence of an additional negative feed-back loop.

## 4 Discussion

Naïve T cell production is highest in young individuals and declines as thymic involution progresses with age. This impaired T-cell production leads to weakened immune responses especially against novel viral infections and increased incidence of autoimmune diseases.

In our previous work age-related down-regulation of Wnt-4 has been identified as a trigger for EMT and pre-adipocyte differentiation during thymic involution (Kvell et al., 2010). In the present work those Wnt receptor associated molecular mechanisms were investigated that can lead to structural and functional decline of the thymic epithelial network.

As in the mouse down-regulation of Wnt-4 expression is known to have occurred by the age of 12 months (Kvell et al., 2010), an earlier time-point was probed. At the age of 9 months Wnt-4 and FoxN1 decline was measured to be moderate (data not shown) along with further characteristic morphological and molecular changes. Interestingly, both Wnt-4 receptors, Fz-4 and Fz-6 are up-regulated at this age coinciding with increased receptor signalling. Apart from increased activation of the inhibitory receptor Fz-6, the expression of CTGF a recently identified potential negative regulator of canonical Wnt signalling is also up-regulated. It has been described that Fz-6 receptor activation can lead to increased kinase (NLK and TAK) activity resulting in phosphorylation of TCF (Luo et al., 2004), an important component of the β-catenin-TCF transcription complex. Once phosphorylated, TCF can no longer bind to β-catenin therefore gene transcription initiated via the canonical Wnt signalling pathway (for example via Fz-4) is inhibited. In contrast to Fz-6 signalling, CTGF uses a different way to interfere with β-catenin dependent signal transduction. CTGF can interact with Fz-8 as well as LRP6, an important co-receptor of Wnt signalling (Mercurio et al., 2004) and can trigger activation of GSK3β (Crean et al., 2004) via PKC ζ. Activation of GSK3β leads to increased phosphorylation and accelerated degradation of β-catenin which results in suppression of canonical Wnt signals.

**Fig. 6:**
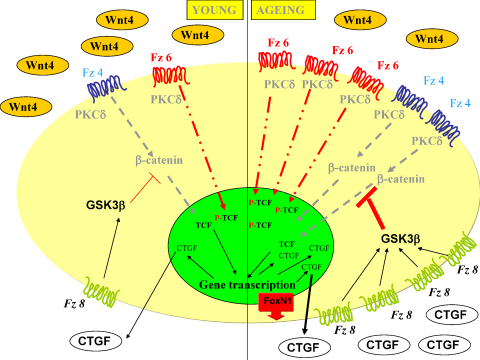
Molecular model of thymic epithelial senescence. In young thymi Wnt-4 levels are high and both Fz-4 and Fz-6 receptors are expressed. Wnt-4 levels decrease with age, while receptor and receptor associated signalling molecule expressions increase triggering multiple suppression pathways of Wnt signalling.

Additional to increasing inhibitory pathways the loss of Wnt-4 levels can amalgamate age related decrease of FoxN1 and therefore loss of TEC identity that eventually leads to EMT and adipoid involution of the thymus. Our current hypothesis describing multiple mechanisms that lead to suppression of Wnt signalling is summarized in Fig. 6.

While it appears that active β-catenin signalling is absolutely essential for the maintenance of thymic epithelium, it is still not clear how imbalance in Wnt signalling affects de-regulation of thymic morphology. This is of note as recent experimental data where prolonged canonical Wnt signalling was enforced in TECs (Zuklys et al., 2009) describes similar changes in the thymic structure that is observed during both physiological and steroid induced ageing of the thymic epithelial network (Talaber et al., 2011). Importance of the balance in Wnt signalling is particularly well demonstrated in a Klotho deficient mouse model (Liu et al., 2007). The secreted protein Klotho can interact with various Wnt family members and suppresses biological activity of Wnts. Tissues and organs from Klotho-deficient animals show evidence of increased Wnt signalling resulting in accelerated cellular senescence both in vitro and in vivo. Based on the above model it has been postulated that depletion of the organ specific stem cell pool might be responsible for the accelerated ageing process in this setting.

Nevertheless, our studies focusing on the role of PKCs in Wnt associated receptor signalling have highlighted additional complexities at the intracellular level. In previous studies PKCα and PKCδ have been implicated to play an active role in disassembly of the nucleus in early apoptosis by phosphorylating nuclear lamin proteins (Dreger et al., 1999). Lamina-associated polypeptide 2 (LAP2) has been described to require phosphorylation via PKCs to fulfil its physiological function and LAP2α is up-regulated during ageing within TECs. While theoretically PKCδ could have a role within the nucleus during senescence, our current experiments support PKCδ activity in the cell membrane as transducer of negative Wnt signals. These findings support our earlier conclusions, namely thymic involution is not directly associated with PKCδ–dependent mass apoptosis and replacement of TECs by invading adipocytes, but rather a slow EMT process that results in adipocyte type trans-differentiation of resident TECs (Kvell et al., 2010; Youm et al., 2009).

Upon Wnt-4 signalling PKCδ seems to associate both with Fz-4 and Fz-6, latter being an inhibitory receptor of the canonical Wnt pathway. If Wnt-4 triggers CTGF gene transcription as a canonical target via Fz-4, then suppression of Fz-6 signalling by down-regulating PKCδ levels can up-regulate CTGF expression (Fig. 3a and b). Additionally, slight down-regulation of CTGF in the presence of PKCδ up-regulation also suits this hypothesis (Fig. 3a). Yet, there are still questions to be addressed. For example as PKCδ can also associate with Fz-4 and have other functions in intracellular signalling therefore the mere up-regulation of PKCδ would not halt β-catenin dependent signal transduction.

Further studies, however, are required and are on their way to identify the role of PKCδ in Fz-6 signalling and their combined task in thymic atrophy using in vitro cell line and in vivo transgenic animal studies.

## 5 Author contribution

ZV, KK and JEP have designed and performed experiments. ZV, KK and GM have participated in sub-cloning of Wnt-4 and PKCδ into viral plasmids, viral vector preparation, establishment of transgenic cell lines, performed Wnt-4 treatment and microarray experiments under the supervision of JEP. Also, ZV, KK and GT performed TEC purification, cDNA preparation and RT-PCR. ZV also performed microarray data analysis, PKCδ activity assays, western blotting and immunoprecipitation under the supervision of JEP. JEP and KK supervised the analysis of Q-RT-PCR data, VC and DB performed statistical analysis. JEP, GA and EJJ helped with the analysis of histology data. All authors discussed the results and commented on the manuscript.

## Figures and Tables

**Table 1:** PCR primers.

Gene	Accession nos.	Forward primer	Reverse primer
β-actin	NM_007393	TGG CGC TTT TGA CTC AGG A	GGG AGG GTG AGG GAC TTC C
Wnt-4 cloning primers	NM_030761	gaagatcttc ATGAGTCCCCGCTCGTGC	ccgctcgagcgg TCATCGGCACGTGTGCAA
Wnt-4 PCR primers	NM_030761	CTC AAA GGC CTG ATC CAG AG	TCA CAG CCA CAC TTC TCC AG
CTGF	NM_010217	GGCCTCTTCTGCGATTTCG	CCATCTTTGGCAGTGCACACT
PKC-δ PCR primers	NM_011103	AGGCCGTGTTATCCAGATTG	CGGTTCATGGTTGGAAACTT
Frizzled 4	NM_008055	TCTGCTTCATCTCCACCACCTT	GCGCTCAGGGTAAGAAAACCT
Frizzled 6	NM_008056	GCGGCGTTTGCTTCGTT	CACAGAGGCAGAAGGACGAAGT
Frizzled 8	NM_008058	TTCCGAATCCGTTCAGTCATC	GCGGATCATGAGTTTTTCTAGCTT
