# Supplementary material for: Multiple suppression pathways of canonical Wnt signalling control thymic epithelial senescence
Source: Mech Ageing Dev. 2011 May;132(5-19):249–56. doi: 10.1016/j.mad.2011.04.007 (PMC3146701; doi:10.1016/j.mad.2011.04.007)
Supplement: Supplementary file 1 [file mmc1.doc]

**Table 1s. Microarray**

| **Accession No.** | **Gene name** | **Fold change** |
| --- | --- | --- |
| [NM_010199.2](http://www.ncbi.nlm.nih.gov/entrez/viewer.fcgi?val=NM_010199.2) | fibroblast growth factor 12 | 2.02 |
| [NM_001122736.1](http://www.ncbi.nlm.nih.gov/entrez/viewer.fcgi?val=NM_001122736.1) | insulin-like growth factor 2 | 2.03 |
| [NM_008871.2](http://www.ncbi.nlm.nih.gov/entrez/viewer.fcgi?val=NM_008871.2) | serine peptidase inhibitor | 2.23 |
| [NM_011941.3](http://www.ncbi.nlm.nih.gov/entrez/viewer.fcgi?val=NM_011941.3) | mitogen activated protein kinase binding protein 1 | 2.25 |
| [NM_008008.4](http://www.ncbi.nlm.nih.gov/entrez/viewer.fcgi?val=NM_008008.4) | fibroblast growth factor 7 | 2.99 |
| [NM_009818.1](http://www.ncbi.nlm.nih.gov/entrez/viewer.fcgi?val=NM_009818.1) | catenin (cadherin associated protein), alpha 1 | 3.49 |
| [NM_008306.4](http://www.ncbi.nlm.nih.gov/entrez/viewer.fcgi?val=NM_008306.4) | N-deacetylase/N-sulphotranspherase 1 | 3.89 |
| [NM_133662.2](http://www.ncbi.nlm.nih.gov/entrez/viewer.fcgi?val=NM_133662.2) | immediate early response 3 | 4.39 |
| [NM_010217.2](http://www.ncbi.nlm.nih.gov/entrez/viewer.fcgi?val=NM_010217.2) | connective tissue growth factor | 6.36 |
| [NM_010499.4](http://www.ncbi.nlm.nih.gov/entrez/viewer.fcgi?val=NM_010499.4) | immediate early response 2 | 6.923 |
| [NM_001085390.1](http://www.ncbi.nlm.nih.gov/entrez/viewer.fcgi?val=NM_001085390.1) | dual specificity phosphatase 5 | 8.44 |
| [NM_009344.3](http://www.ncbi.nlm.nih.gov/entrez/viewer.fcgi?val=NM_009344.3) | pleckstrin homology-like domain | 11.93 |
| [NM_010234.2](http://www.ncbi.nlm.nih.gov/entrez/viewer.fcgi?val=NM_010234.2) | FBJ osteosarcoma oncogene | 18.10 |
