# Supplementary material for: Multiple suppression pathways of canonical Wnt signalling control thymic epithelial senescence
Source: Mech Ageing Dev. 2011 May;132(5-19):249–56. doi: 10.1016/j.mad.2011.04.007 (PMC3146701; doi:10.1016/j.mad.2011.04.007)
Supplement: Supplementary file 2 [file mmc2.ppt]

## Slide 1
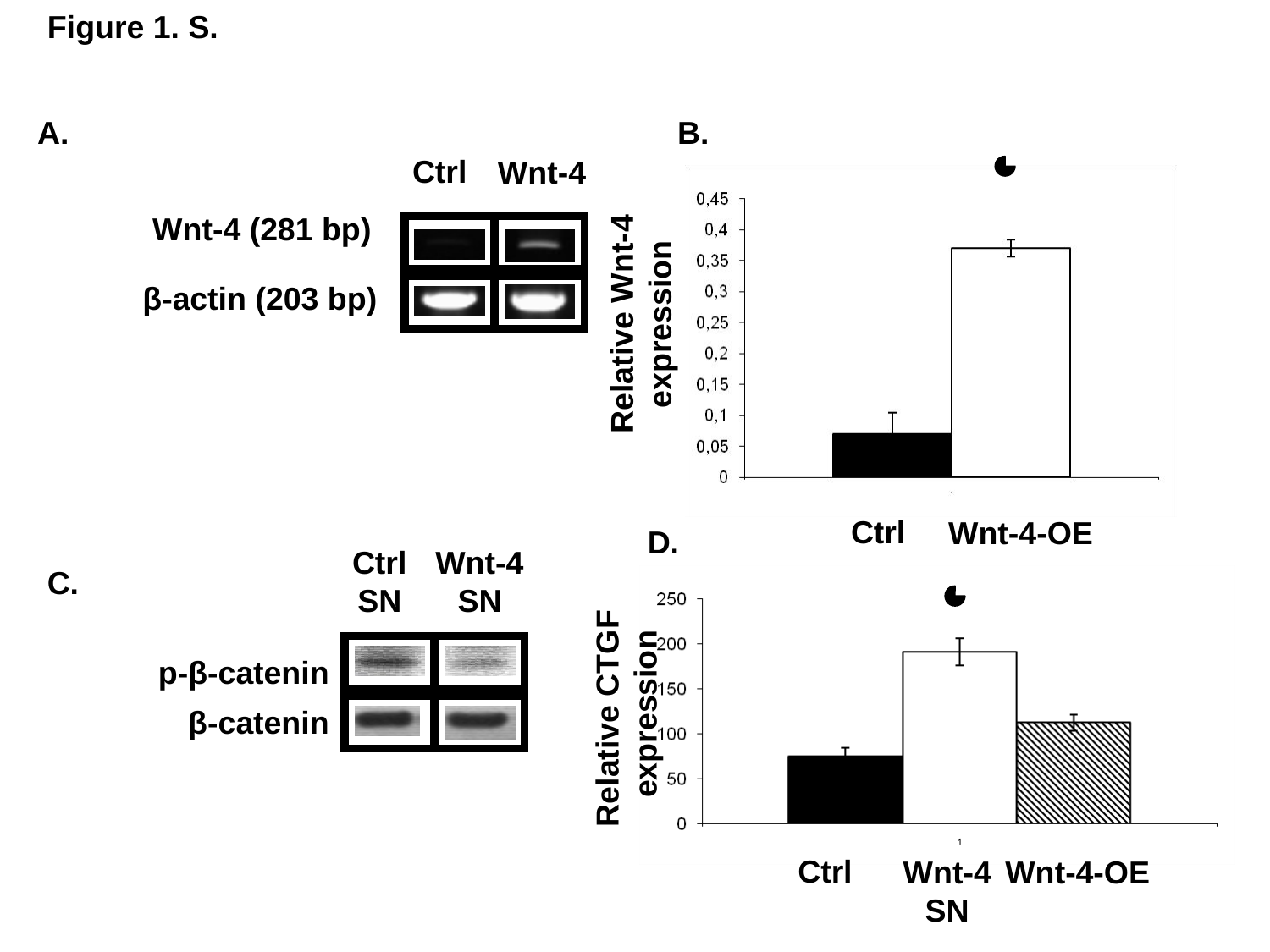

Figure 1. S.
A.
B.
Ctrl
Wnt-4
Wnt-4 (281 bp)
β-actin (203 bp)
Relative Wnt-4
expression
Ctrl
Wnt-4-OE
D.
Ctrl SN
Wnt-4 SN
C.
p-β-catenin
Relative CTGF
expression
β-catenin
Ctrl
Wnt-4
SN
Wnt-4-OE

## Slide 2
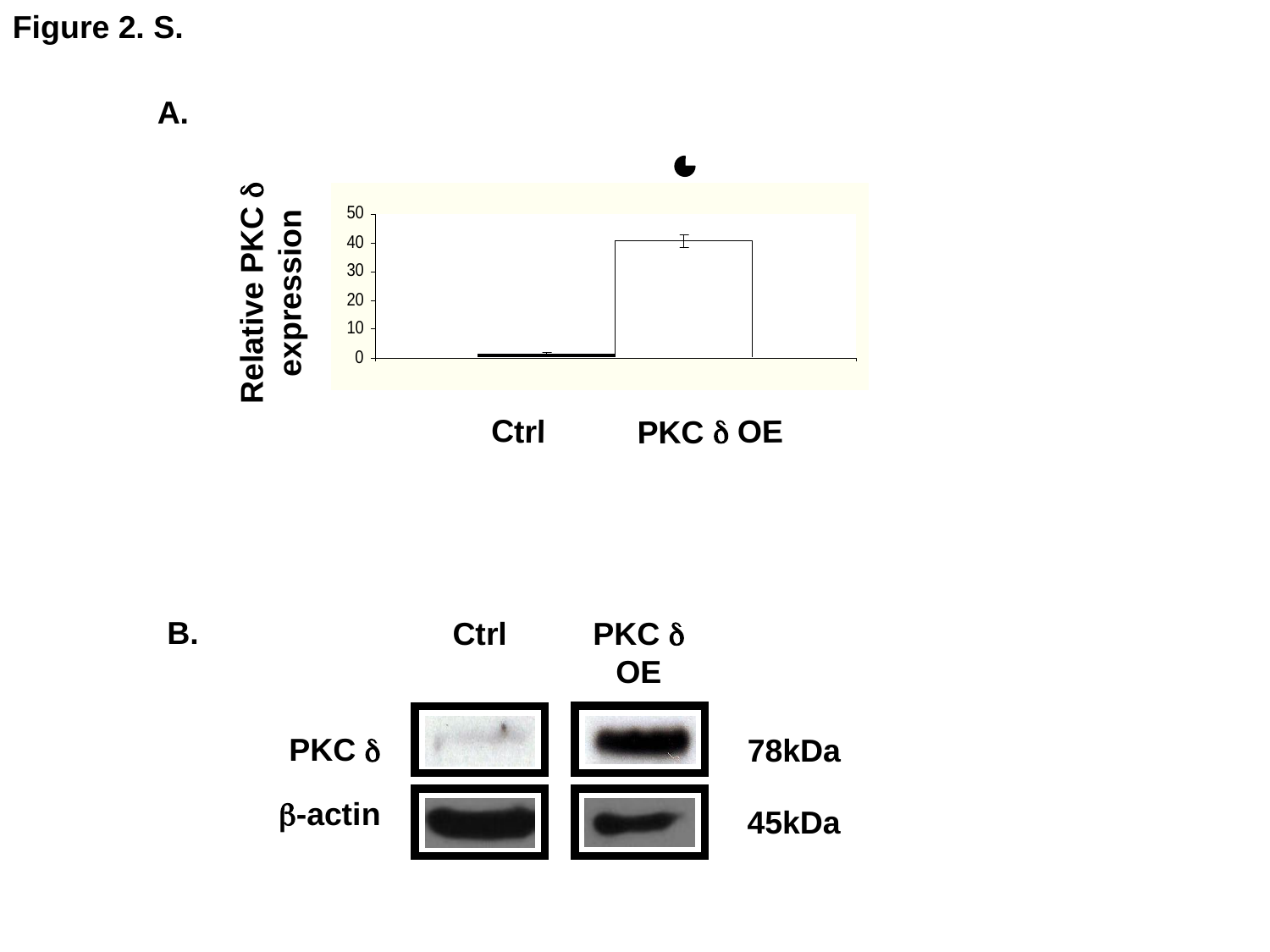

Figure 2. S.
A.
Relative PKC 
expression
Ctrl
PKC  OE
B.
Ctrl
PKC  OE
PKC 
78kDa
β-actin
45kDa

## Slide 3
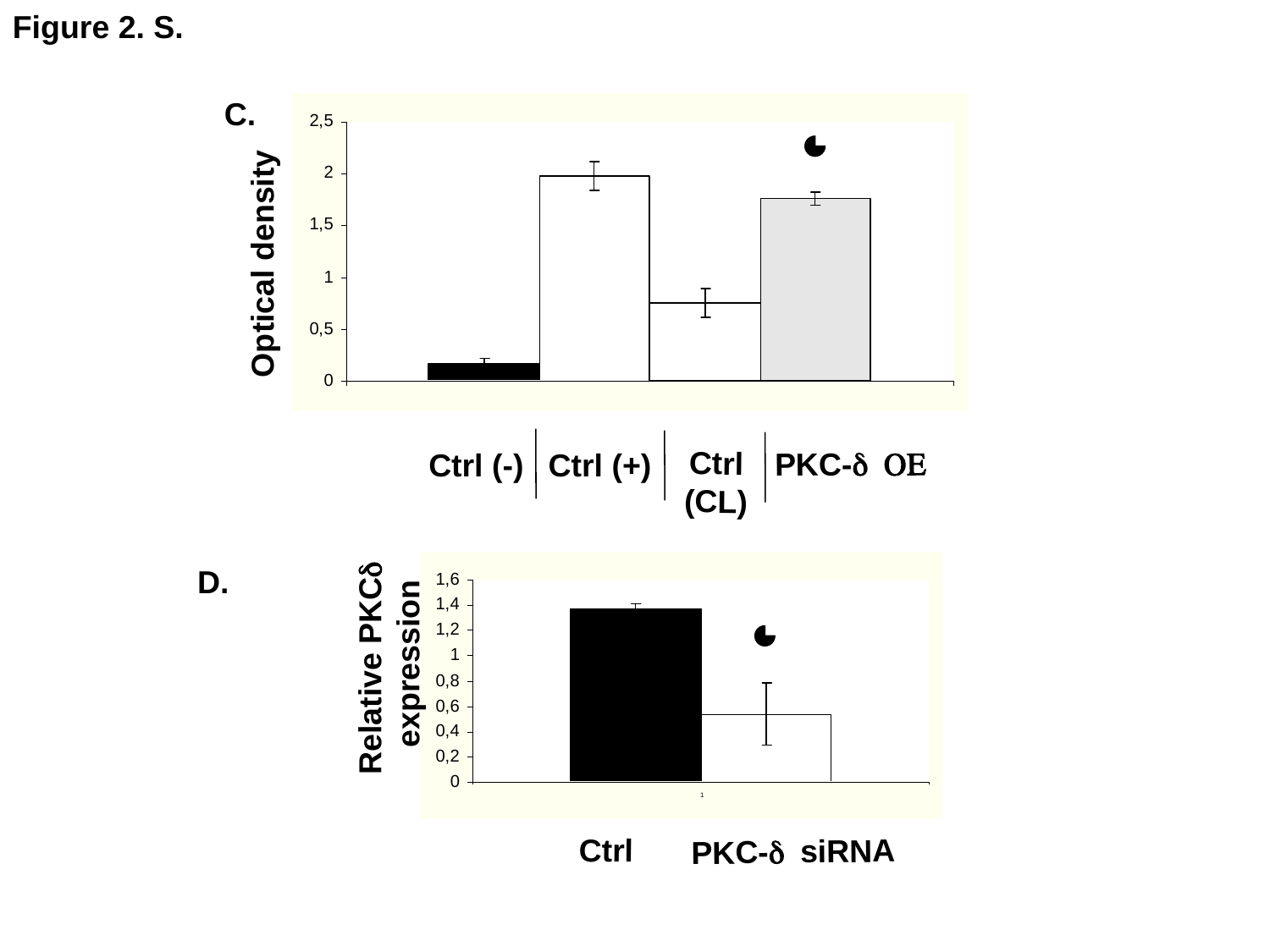

Figure 2. S.
C.
Optical density
Ctrl
(CL)
PKC- 
Ctrl (-)
Ctrl (+)
D.
Relative PKCδ
expression
Ctrl
PKC- siRNA
