# Supplementary material for: Multiple suppression pathways of canonical Wnt signalling control thymic epithelial senescence
Source: Mech Ageing Dev. 2011 May;132(5-19):249–56. doi: 10.1016/j.mad.2011.04.007 (PMC3146701; doi:10.1016/j.mad.2011.04.007)
Supplement: Supplementary file 3 [file mmc3.doc]

**Supplementary figures:**

**Supplementary Fig. 1**. Generation and analysis of Wnt-4 over-expressing transgenic Tep1 cell line. (A) RT-PCR analysis of Wnt-4 over-expressing transgenic cells compared to mock (GFP) transfected TEP1 cells. (B) Q-RT-PCR analysis of Wnt-4 expression in Wnt-4-GFP over-expressing compared to GFP transfected TEP1 cells. (C) Verification of Wnt-4 functionality by western blot analysis of p-β-catenin and total β-catenin levels. (D) Q-RT-PCR analysis of CTGF expression in control, Wnt 4-treated, Wnt-4 over-expressing Tep1 cells. Statistically significant differences are marked by asterisks.

**Supplementary Fig. 2.** Generation and analysis of PKCδ over-expressing and PKCδ siRNA silenced transgenic Tep1 cells. (A) PKCδ expression by Q-RT-PCR in control and PKCδ over-expressing Tep 1 cells. (B) PKCδ expression by Western blot using control and PKCδ overexpressing Tep1 cells. (C) PKCδ activity was also measured by colorimetric ELISA assay, relative absorbance values of controls, GFP only and PKCδ over-expressing cells are shown. (D) PKCδ specific RNAi silencing measured by Q-RT-PCR in mock and PKCδ specific siRNA-transfected TEP1 cells. Statistically significant differences are marked by asterisks.
